# Evaluation of Residual Diazinon and Chlorpiryfos in Children Herbal Medicines by Headspace-SPME and GC-FID

**Published:** 2014

**Authors:** Mohammad Hossein Mosaddegh, Fakhrossadat Emami, Gholamreza Asghari

**Affiliations:** a *Pharmacology and Toxicology Department, Medicine Faculty, Yazd Shahid Sadoughi University of Medical Sciences, Yazd, Iran. *; b *Isfahan Pharmaceutical Sciences Research Center, Isfahan University of Medical Sciences, Isfahan, Iran.*

**Keywords:** Diazinon, Chlorpyrifos, SPME (Solid-Phase Microextraction), GC-FID, Children herbal medicine

## Abstract

The oldest method for the managing of the illness is the use of medicinal plants. The use of herbal products as the first choice in self-treatment of minor conditions continues to expand rapidly across Iran. This makes the safety of herbal products an important public health issue.

Pesticides are used widely in agriculture to increase the production by controlling the harmful insects and disease vectors, however it has some hazards on biological system of human especially children. The present study was designed to examine the residual amount of organophosphorus pesticides (Diazinon and Chlorpyrifos) in children herbal medicines available in the Iranian market. Five children herbal medicine liquid dosage forms were purchased from pharmacy store. They were extracted with SPME (Solid Phase Microextraction) using the PDMS-DVB fibre. Then the extracts were injected into a GC. The gas chromatograph was Younglin model YL 6100 equipped with a flame ionization detector. The column was Technokroma 60 m length, 0.53 mm internal diameter and 1.25 µm film coated. The presence and quantity of Diazinon and Chlorpyrifos were evaluated using their standard curves. Trace amounts of chlorpyrifos and diazinon were detected in a few herbal medicines. Based on European pharmacopeia, threshold limits of chlorpyrifos and diazinon residues for medicinal plant materials are 0.2 and 0.5 mg/Kg, respectively. Our analysis results showed that residue limits of these two pesticides in five children herbal medicines are ignorable.

## Introduction

Herbal products include a range of self-prescribed preparations with plant origins. They commonly characterized as foods, supplements, cosmetics and medicinal products. The use of herbal medicines has a long history in the treatment of diseases all over the world and still makes an important part of now-days medicine. However, herbal medicines have some drawbacks such as contamination with chemicals (1).

One of the most contaminants of herbal medicine is pesticide (1). Farmers used Pesticides widely to increase the herbal production by control of the harmful insects. The wide spread use of pesticides in agriculture has caused severe environmental pollution and possible health hazards including severe acute and chronic cases of human poisonings. World Health Organization estimates that the incidence of pesticide poisonings in developing countries has doubled during the past decade (2). Among pesticides, organophosphates are usually used as insecticides, and are generally the most toxic pesticide. Residual amounts of Organophosphate pesticides have been detected in the soil, water, vegetables, grains and other food products. Because of the wide availability of Organophosphates, toxic effects in human being have been shown (3).

The primary molecular mechanism of action of the organophosphates is binding with acetyl cholinesterase (AChE) enzyme and so inhibition of AChE, which results in accumulation of acetylcholine (3,4 and 5). Toxicity of Organophosphates also causes adverse effects on many organs such as immune system, urinary system and reproductive system. The other effects are hematological and biochemical changes (2, 6).

Organophosphates can induce oxidative stress and produce free radicals in biological systems (7). Animal studies show that prenatal or postnatal exposure of rodents to chlorpyrifos and diazinon causes considerable changes in a range of biochemical, morphological and cellular parameters related to nervous system development, leading to persistent defects in behavior and cognition (8). Another study determined that children had neurological and developmental defects after indoor application of organophosphates (9).

Diazinon and chlorpyrifos are commonly used thionophosphorous organophosphate pesticides (4,10). They can be absorbed through the digestive system, the skin, and respiratory tract (2). Diazinon is a very highly toxic organophosphate compound (4). Chlorpyrifos at low concentrations can interfere with calcium metabolism; in fact, skeletal malformations were mainly happened in a dose dependent manner (11).

An important public health task has been the need to keep children health. To achieve this goal, the scientific community needs risk assessment methods scientifically for children (12). Recent studies show that young children could expose to pesticides during oral exploration of their environment and dermal contact with floors and other surfaces (13). 

For the pesticides extraction, several methods are available. The common extraction method is liquid–liquid extraction. Its advantages are simplicity and application easiness, but it requires time, large sample volumes and uses a large volume of organic solvents. Another more recent method is solid phase extraction (SPE). Its advantages are consuming much less organic solvents and to be automatable, but require considerable equipment and extraction cartridges, which are not reusable. These two methods extract other impurities, which can cause an important background noise (14, 15). 

The most recent extraction method from liquid samples is solid-phase micro-extraction (SPME). It is an extraction method without organic solvent, and does not require a complicated apparatus. The analytes are adsorbed on the SPME fiber and then desorbed in the injection port of a gas chromatograph. SPME theoretical bases have been widely detailed (15, 16 and 17), as well as different applications in the fields of toxicology, environment, pharmacy and food (18, 19 and 20). 

SPME-GC method is consist of sorption of analytes on a fiber, their desorption in a GC injection port, separation in a chromatographic column, detection and quantification (21, 22). SPME combines sampling and concentration of analytes in a single step (23). HS-SPME-GC-FID revealed to be a clean, simple, fast and reliable method (24). In theory, the HS-SPME has several advantages: the fibers not exposed to the sample, the background noise and matrix effects are reduced, which the lifetime of SPME fiber be enhanced (25).

There are several reports on contamination of herbal medicines with pesticides (1,26,27,28 and 29). The present study evaluated the residual amount of organophosphorus pesticides (Diazinon and Chlorpyrifos) in children herbal medicines available in the Iranian market.

## Experimental

*Chemicals and material*

Children herbal medicines were prepared from market, their batch numbers and the plants made formulation were recorded (Table 1):

Stock solutions containing 100 mg per mL of standards of diazinon and chlorpyrifos were prepared in methanol. Sodium chloride, sodium hydroxide (NaOH) and hydrochloric acid (HCl) were analytical grade and were obtained from Sigma. They were used to prepare saturated sodium chloride and 0.1 M HCl and NaOH solutions. Diazinon and Chlorpyrifos as standards were obtained from Sigma Aldrich, UK. Headspace vials (15 mL amber glass) with septum caps were heated and agitated with a hot plate stirrer. A manual assembly for SPME, with replaceable 75-μm PDMS-DVB coated extraction fiber coated with polydimethylsiloxane/divinylbenzene, was obtained from Sigma-Aldrich Company *Ltd*, UK. Prior to use, the fiber was exposed to a temperature of 250 °C in the GC injection port overnight to remove contaminants as well as conditioning the fiber.

**Table 1:** Children herbal medicines used in this study

**Name**	**Dosage form**	**Pharmacology effects**	**Components**	**Active ingredients**
Gol-Grip	oral solution	Carminative	*Anethumgraveolens*, sodium bicarbonate	Carvon, limonene, bicarbonate sodium.
Senagol	Syrup	Laxative	*Cassia obovata* extract	anthraquinones,sennosides A, B,C ,D,mucilage,flavenoides
Caraway mixture	oral solution	Carminative	*Carumcarvi, Foeniculumvulgare, Menthaarvensis, Menthalongifolia* extracts	carvon,anethol, fenkon,menthol,menthon
Thymian	Syrup	antitussive	*Thymus vulgaris ,Foeniculumvulgare, Eucalyptus globulus, Saturejahortensis extracts, glycerizic acid.*	Flavnoides,thymol,anethol,fenkon,carvacrol
Children Tussian	Syrup	Antitussive in bronchitis and whooping cough	*Thymus vulgaris* extract	thymol,carvacrol

*Instrumentation*

The analysis was performed first on a gas chromatograph fitted with a flame ionization detector (GC-FID). Helium was the carrier gas, with a flow rate of 1 mL/min. The gas chromatograph was Younglin model YL 6100 equipped with a flame ionization detector. The column was Technokroma; 60 m length, 0.53 mm internal diameter and 1.25 µm film coated. Standard solutions were injected splitless with the split valve closed for 2 min and the injector port at 250 °C. The oven temperature was held at 80 °C for 2 min and subsequently ramped at a rate of 5 °C/min until 270 °C and held there for 5 min. The SPME injection was performed with desorption time of 2 min at 250 °C by closing the split valve for 2 min. The fiber was kept in the injection port for a further 2 min to ensure all compounds have been desorbed from it. The detector was maintained at 280 °C. Helium gas flow was 300 mL/min. Hydrogen gas flow was 30 mL/min.

## Results

*Optimization of HS-SPME-GC-FID methodology*

*Fiber selection and fiber affinity for the pesticides*

**Figure 1:**
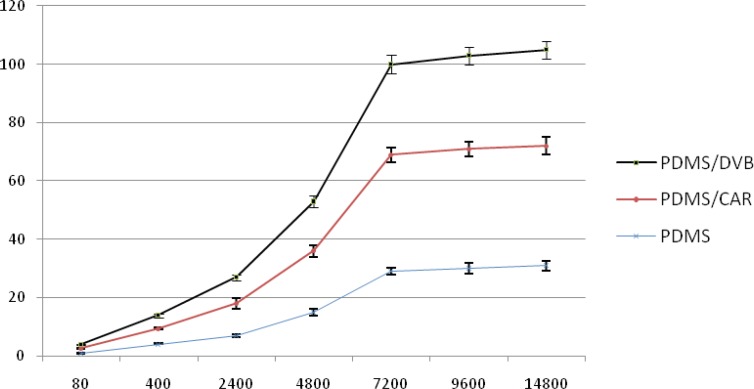
Comparison of different SPME fibers used for analysis of diazinon

In this study, three types of SPME fiber coatings (PDMS, PDMS-DVB, PDMS-CAR) were compared for their ability to extract pesticides from the headspace. Diazinon was used as the representative organophosphorus pesticide in evaluating of fibers in the extraction of pesticides. A graph of area response for three fibers was plotted. The optimum fiber for sampling was determined. According to the Figure 1, PDMS-DVB fiber was selected in extraction of these pesticides because of more affinity for absorbing pesticide in comparison to others.

*Extraction time optimization*

**Figure 2:**
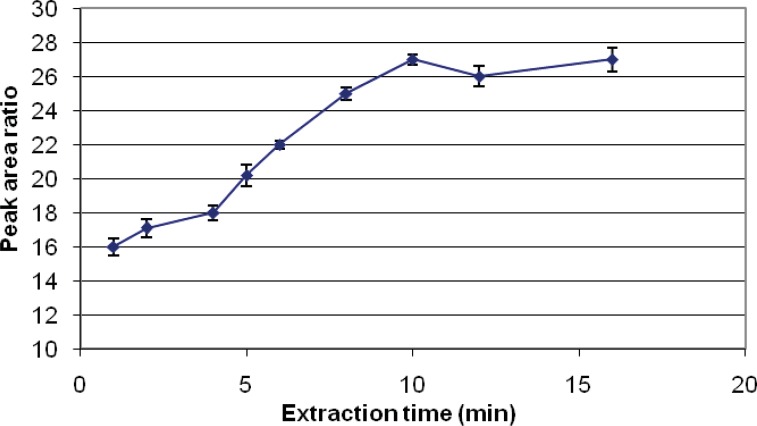
Extraction time used for analysis of chlorpyrifos and diazinon

The first step and the most important is the determination of the required time to reach equilibrium for the partitioning of the analyte between the aqueous phase and the SPME coating. A graph of area response against extraction time was plotted and the suitable extraction time for analysis of this pesticide was established accordingly. Due to the similarity of pesticides studied, this adsorption period was employed for all this study. Using DVB-PDMS fiber, the areas for diazinon increased with an increase from 1 to 10 min of the extraction time, while no significant changes were observed with increasing the time of extraction to 16 min (Figure 2). Therefore, 10 min of extraction with DVB/PDMS fiber coating was chosen as the optimal extraction time for the analysis of diazinon and chlorpyrifos by HS-SPME.

*Desorbing time optimization*

**Figure 3:**
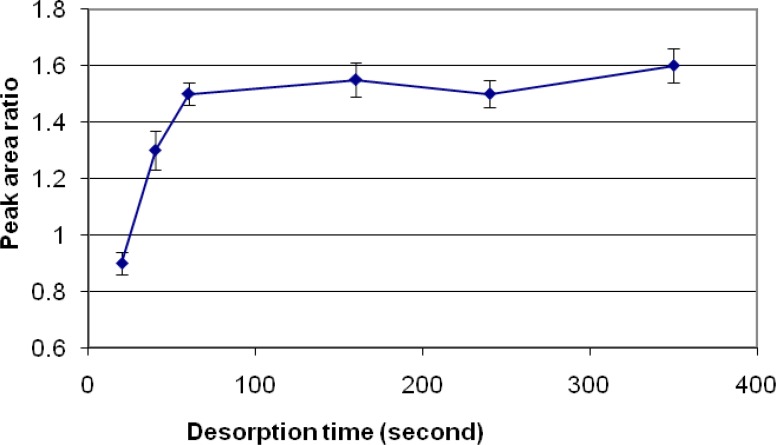
Desorption time optimization used for analysis of chlorpyrifos and diazinon

Samples extracted with DVB/PDMS fiber were subjected to injection port at 250 °C at different times, ranging from 20 to 350 seconds. A graph of area response vs. desorption time was plotted. The areas for diazinon increased with an increase from 20 to 160 seconds of desorption time, while no significant changes were observed with increasing the time of desorption to 350. These results show that the optimum time for desorbing of these pesticides is 160 seconds (Figure 3).

*PH optimization in extraction*

**Figure 4:**
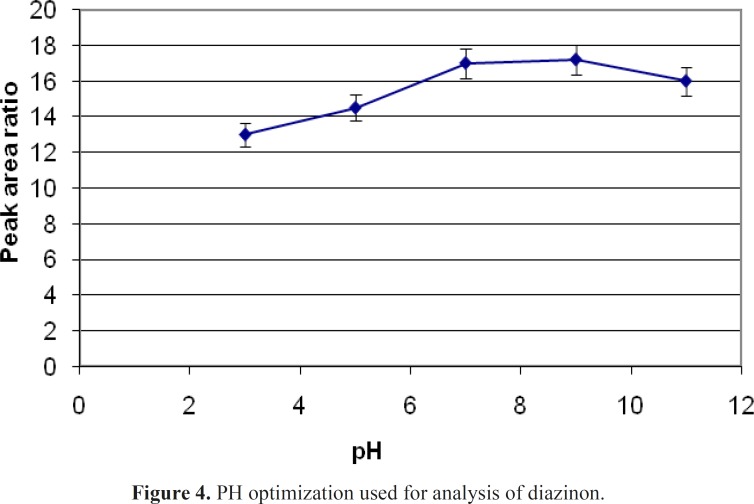
PH optimization used for analysis of diazinon

If the PH of sample changed, the ionization potency of an analyt in water would be changed that can effects on the mount of sample extraction 5 mL of spike with definite concentration of diazinon in different pH of 3,5,7,9 and 11 have experimented. NaOH and HCl were used for changing of pH. A graph of area response vs. pH was plotted. The areas for diazinon improved with an increase of pH from 3 to 7, while no significant changes were observed with increasing PH to11. These results show that optimum pH in this study is 7 (Figure 4).

*Selection appropriate volume of sample*

**Figure 5:**
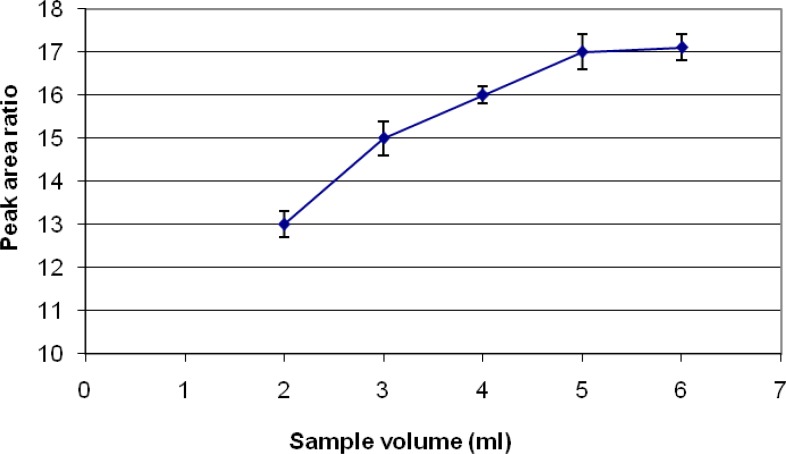
Sample volume selection used for analysis of diazinon

Samples spiked with definite concentration of diazinon standards were subjected to extraction at different volumes, ranging from 2 to 6 mL. A graph of area response vs. volume of sample was plotted. The optimum volume for sampling that determined is 5 mL (Figure 5).

*Salting out effects*

**Figure 6:**
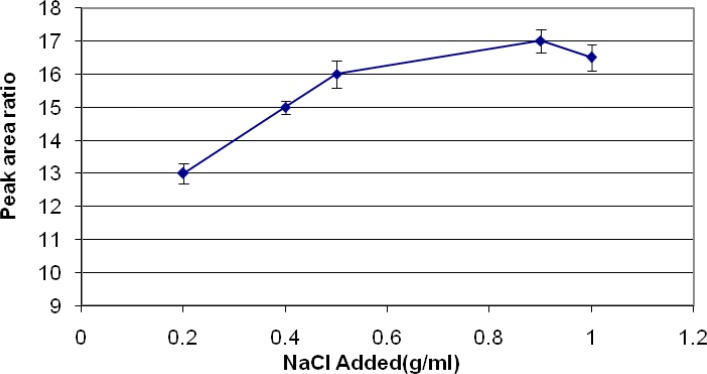
Salting out effect on analysis of diazinon

Using of NaCl about 0.2-1 gram in each ml of samples were evaluated. A graph of area response vs. using of NaCl was plotted. The graph shows that using of 0.9 g/mL NaCl can improve the quality of extraction (Figure 6).

*Calibration curves of diazinon and chlorpyrifos by HS-SPME-GC-FID*

Stock standard solutions of chlorpyrifos and diazinon, 10 µg/mL (10ppb), were prepared. A series of chlorpyrifos standard solutions at 0.8, 1.2, 2 and 3.6 µg/mL concentrations were prepared by dilution of the chlorpyrifos stock solution. 

**Figure 7:**
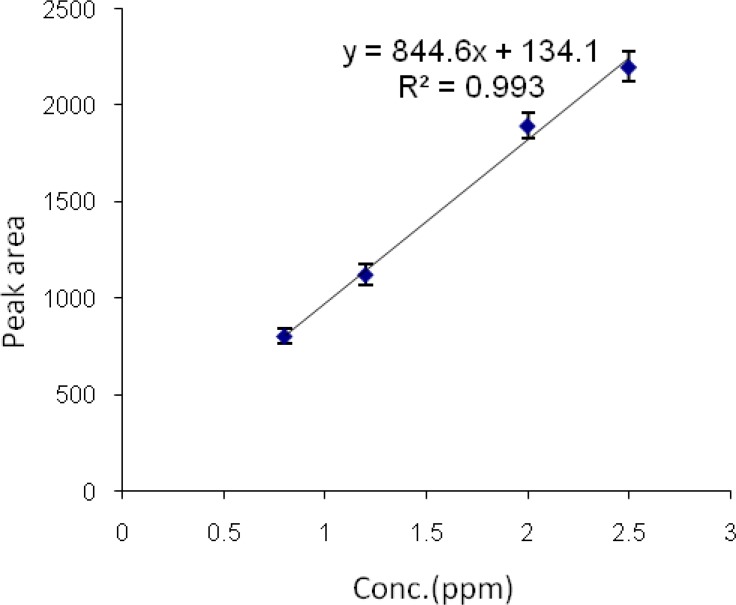
Chlorpyrifos calibration curve

**Figure 8:**
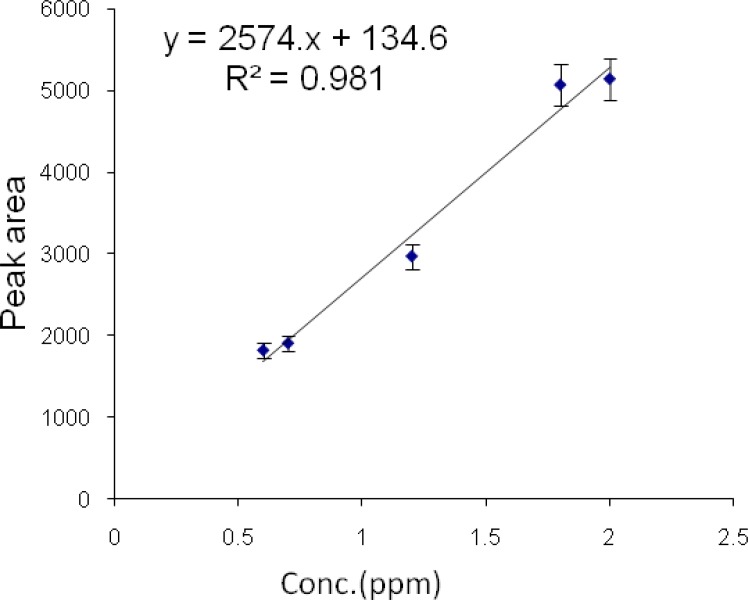
Diazinon calibration curve

Then a series of diazinon standard solutions at 0.6, 0.7, 1.2, 1.8 and 2 µg/mL concentrations were prepared by dilution of the diazinon stock solution. Then 0.9 g/mL NaCl was added to each sample to improve the extraction yield. Each sample was then shaken for 5 min and allowed to equilibrate. Each sample was extracted by headspace SPME at the temperature of 60 °C for 10 min. All analyses were performed in triplicates. Graphs of area response vs. concentration of standard diazinon and chlorpyrifos were plotted. The calibration curve and linear range of headspace SPME extraction of chlorpyrifos and diazinon were determined (Figures 7 and 8).

The limits of detection of chlorpyrifos and diazinon were 80 and 60 ηg/mL, respectively. Retention times for chlorpyrifos and diazinon were 4.44 and 5.58 min, respectively. The method was linear in the range 0.8-3.6 µg/mL and 0.6-2 µg/mL for chlorpyrifos and diazinon, respectively. The correlation coefficients (r^2^) were 0.993 and 0.981 for chlorpyrifos and diazinon, respectively. 

## Discussion

The request for herbal medicines increased in recent years, and people usually are changing from synthetic pharmaceutical drugs to the naturally based products.

Pesticides largely use on crops to protect them against various pests. Herbal medicines, which make from infusions, decoctions, tinctures and essential oils, contain pesticides. This fact triggered so many scientific investigations to assess the health hazards more precisely. 

Consumers of herbal medicines are anticipating these trade products have no residues of pesticides. Now days, the extensive cultivation of medicinal plants is not possible without using pesticides. Diazinon and chlorpyrifos are commonly used thionophosphorous organophosphate pesticides, which absorb through the digestive system, the skin, and respiratory tract (2, 4 and 10). An important public health task is keeping children health. Recent studies show that young children could expose to pesticides during oral exploration of their environment and dermal contact with floors and other surfaces (13). The aim of the present research was to determine the residues of chlorpyrifos and diazinon in some of the trade herbal medicines that prescribe for children.

Different SPME fibers have different abilities to extract chemicals from matrices. The SPME fiber selection for analysts in selecting the appropriate SPME fiber for a certain chemicals presents some difficulties. Among the selected fibers, PDMS/DVB showed more affinity to diazinon, and so we used it. The reduction of time for an analysis is very important. Therefore, the extraction and desorption times using the selected SPME fiber should be determined for an analysis. The optimum extraction and desorption times for this study were 10 min and 160 min, respectively. Whereas, the ionization of analytes can determine the extraction yield, the optimum pH for extraction of diazinon is 7. The optimum sample volume for the headspace extraction of diazinon is 5 mL. 

Reduction of the solubility of diazinon in the aqueous phase can increase the migration of diazinon to headspace and so the amount of analyte extracted by the fiber, which can do by addition of NaCl. The optimum concentration of NaCl for diazinon extraction was 0.9 g/mL. 

There are several reports from different countries on determination of pesticide residues in herbal medicines. A study in Egypt found malathion, dimethoate and profenofos in most herbal samples. Malathion and chlordane were with the highest mean level of 0.694 and 0.212 mg/Kg in chamomile, respectively. The highest level of profenofos was in anise. Aldrin, dieldrin, chlordane and lindane were at the lowest mean levels. Chlorpyrifos, parathion, diazinon and endosulfan were not detectable in most of the samples under investigation. The percentage of samples contaminated with the pesticides ranged from 70 to 100% (28). 

A Brazilian study of samples of passionflower (Passiflora L.) showed organochlorine pesticide residues (dieldrin, lindane, tetradifon, chlorothalonil, and α-endosulfan) at a level of 21 µg/Kg to 71.4 µg/Kg (29). Xue *et al.* detected α-BHC, PCNB, HCH, and tecnazene in 280 traditional Chinese medicine samples (30). Wei Fang *et al*. quantified the amount of organophosphorous pesticides including diazinon in soil by headspace (HS) solid phase microextraction and gas chromatography coupled with flame ionsation detector (31).

In a study in South Korea, twenty-seven pesticides were determined in herbal medicines using GC-MS-SIM (gas chromatograph-mass spectrometer in selection of ion monitoring mode) in electron impact ionization (EI) mode. The limit of detection for organophosphorous pesticides was 0.1 ρg. Chlorpyrifos was only in Zingiberis rhizoma at 0.3 ppm. However, diazinon was in Platicodi Radix, Auranti Nobilis Pericarpium and Lycii Fractus at concentrations of 0.2, 0.03, and 0.1 ppm, respectively (27).

## Conclusion

As our results showed the concentration of diazinon and chlorpyrifos were undetectable using GC-FID. Based on European pharmacopeia, threshold limits of chlorpyrifos and diazinon residues for medicinal plant materials are 0.2 and 0.5 mg/Kg, respectively. In our study, the limits of detection of chlorpyrifos and diazinon were 80 and 60 ηg/mL, respectively. They are below the threshold limits and so they are acceptable in concern with the pesticides residue limits.
